# Negative Factors Influencing Multiple-Trauma Patients

**DOI:** 10.3390/clinpract14040126

**Published:** 2024-08-13

**Authors:** Mihaela Anghele, Virginia Marina, Aurelian-Dumitrache Anghele, Cosmina-Alina Moscu, Liliana Dragomir

**Affiliations:** 1 Clinical-Medical Department, Faculty of Medicine and Pharmacy, “Dunărea de Jos” University, 47 Str. Domnească, 800201 Galati, Romania; mihaela.anghele@ugal.ro (M.A.); lilianadragomir2017@gmail.com (L.D.); 2 Medical Department of Occupational Health, Faculty of Medicine and Pharmacy, “Dunărea de Jos” University, 47 Str. Domnească, 800201 Galati, Romania; 3 Department of General Surgery, Faculty of Medicine and Pharmacy, “Dunărea de Jos” University, 47 Str. Domnească, 800201 Galati, Romania; anghele_aurelian@yahoo.com; 4 Emergency Department, Faculty of Medicine and Pharmacy, “Dunărea de Jos” University, 47 Str. Domnească, 800201 Galati, Romania; cosmina_caluian@yahoo.com

**Keywords:** multiple trauma, Injury Severity Score, intracranial hemorrhage, hematoma

## Abstract

Background and objectives: This study aimed to assess the impact and predicted outcomes of patients with multiple trauma by identifying the prevalence of trauma sustained and associated complications. Materials and Methods: This retrospective cohort study focused on individual characteristics of patients with multiple trauma admitted to our County Emergency Hospital. The final table centralized the characteristics of 352 subjects aged between 3 and 93 years who presented with multiple trauma from 2015 to 2021. Inclusion criteria for this study were the presence of multiple trauma, intervention times, mentioned subjects’ ages, and types of multiple trauma. Results: Patients with multiple trauma face an increased risk of mortality due to the underlying pathophysiological response. Factors that can influence the outcomes of multiple-trauma patients include the severity of the initial injury, the number of injuries sustained, and the location of injuries. Conclusion: The first 60 min after trauma, known as the “golden hour,” is crucial in determining patient outcomes. Injuries to the head, neck, and spine are particularly serious and can result in life-threatening complications.

## 1. Introduction

The term “multiple trauma” is commonly found in the literature referring to injuries involving more than two anatomical segments or organs, with at least one being potentially life-threatening. According to the new Berlin definitions, multiple trauma is defined as an ISS greater than 16 and an AIS greater than 3 for at least two segments. However, an ISS of 6 for only one segment is incompatible with life and produces a total ISS of 75 points [1].

The management of multiple trauma has significantly improved in recent years. Despite these advancements, multiple trauma remains the leading cause of death in individuals under 40 years of age [2,3,4]. The primary causes of traumatic death worldwide are road traffic accidents, followed by suicide and homicide [5].

Immediate and early trauma-related deaths are often due to severe primary brain injury or massive hemorrhage (hemorrhagic shock). Late mortality can result from secondary brain injury and systemic organ failure [6]. Tissue injuries, such as lacerations and contusions, can lead to local tissue hypoxia and hypotension, triggering local and systemic host responses [7]. These responses can cause a systemic inflammatory reaction, which can be exacerbated by secondary trauma like ischemia or reperfusion injury, surgery, or infection [7,8].

The first 60 min after trauma, known as the “golden hour”, are crucial in determining patient outcomes. Definitive trauma care with resuscitation should be initiated during this early window, as it has been emphasized, taught, and practiced globally for over four decades. The primary steps in early trauma management include primary assessment, simultaneous resuscitation, a reassessment of airway, breathing, and circulation, and secondary assessment. Primary assessment provides essential baseline data for patient survival when life or limb is at risk. Resuscitation should begin simultaneously with the primary assessment whenever instability is detected. Secondary assessment should follow the completion of primary assessment and resuscitation [9].

Individuals with multiple trauma require ongoing support and care to address the long-term effects of their injuries on their mental health [10].

The management of multiple trauma requires well-defined protocols, such as ALS (advanced life support), a universal protocol that includes both the assessment and management of trauma patients. ALS includes primary, secondary, and tertiary assessment [11].

The primary purpose of primary assessment is to identify and immediately treat potentially life-threatening conditions. Imaging plays an active role in diagnosing multiple-trauma patients. Computer tomography is preferred since it brings crucial information in a short amount of time. Magnetic resonance imaging is less feasible in multiple-trauma patients because it is a lengthy process and due to other extrinsic limiting factors [12].

The first and most important step in the ALS protocol is airway assessment and management, including managing the cervical spine for protection. In patients with apnea, a GCS (of less than or equal to 8), orotracheal intubation, and mechanical ventilation are required [13].

The second step includes the assessment of breathing and ventilation, where respiratory rate and efficiency are evaluated. Chest assessment is also necessary to identify potentially life-threatening trauma. Critical conditions requiring immediate management, which may be complicated by hypoxia, hypovolemia, a low heart rate, and even “exitus letalis”, include tension pneumothorax, massive closed hemothorax, cardiac tamponade, and open hemothorax rib flap [14].

One of the most common complications encountered in multiple trauma is massive hemorrhage, with the most common reason for shock in these patients being hypovolemic shock resulting from massive hemorrhage. Hemorrhagic shock is characterized by tachycardia, hypotension, increased capillary refill time, increased respiratory rate, and altered mental status [15].

In the case of multiple-trauma patients, it is necessary to remove clothing at the scene of the accident to conduct a comprehensive assessment. Therefore, another important step in managing these patients is to minimize environmental exposure and consequently minimize heat loss [16].

The early identification of life-threatening injuries that require immediate management and other injuries that may become life-threatening if not properly managed are primary steps in minimizing the negative impact on patients with multiple trauma.

## 2. Materials and Methods

This retrospective cohort study followed the individual characteristics of patients with multiple trauma admitted to the Galati County Emergency Hospital. The final database was developed using information collected from observation sheets of patients who met the inclusion criteria for this study.

The centralized table included data on 352 subjects, aged between 3 and 93 years, who experienced multiple trauma between 2015 and 2021. This table was created and formatted using Microsoft Excel 2019 and then exported to SPSS v26 software.

The final statistical analysis included graphical and descriptive elements, as well as specific statistical measures like mean, standard deviation, minimum/maximum values, kurtosis index, and skewness index.

The study aimed to identify factors that could negatively impact the outcome of patients with multiple trauma.

Inclusion criteria involved the presence of multiple trauma, documented intervention times, subject age, and types of multiple trauma.

The exclusion criteria for the study included patients and their families who refused to participate, as well as patients with isolated traumas that posed a minimal or non-existent risk of complications.

## 3. Results

In the first instance, the objective was to assess the sociodemographic distribution of the group to compare their results with those in the existing literature. After evaluating the age distribution, it was found that the mean age of the group was 29.94 years, with a standard deviation of 14.266. The youngest member was 3 years old, while the oldest was 93. The skewness index was 1.016, indicating a positive skew in the group’s distribution.

Other sociodemographic factors examined included gender and the year of patient registration. Frequency analysis revealed a higher prevalence of males (72.7%) than females (27.3%).

Regarding registration years, the prevalence of multiple trauma in the group by gender and year of case registration is represented in Table 1.

Further analysis identified the prevalence of different types of multiple trauma in the study group. Road traffic accidents were the most common (68.09% of patients), followed by falls from heights (15.50% of patients). Other injuries and their prevalence are listed in Table 2.

To assess the impact and predicted outcomes of patients with multiple trauma, this study identified the prevalence of trauma sustained and associated complications. The most common traumas in this group were traumatic brain injury and traumatic facial injury. The distribution of these traumas, categorized by severity in relation to the total number of patients, was as follows: 13.4% had grade 0 minor acute TBI, 7.1% had grade 1 minor acute TBI, 1. 4% had grade 2 minor acute TBI, 2% had moderate TBI, 20.2% had severe acute TBI, 9.9% had TBI, 5.1% had craniofacial trauma (CFT), and 1.1% had TBI and CFT. Additionally, subarachnoid hemorrhage (SAH) was predominantly traumatic, with only 6.8% of patients having SAH (Table 3).

Most subjects in this study group had no hematomas (90.9%). The types of hematomas identified and their prevalence were as follows: 1.4% had epicranial hematomas, 4.8% had subdural hematomas, 2.3% had extradural hematomas, 0.3% had paravertebral hematomas, and 0.3% had intraparenchymal hematomas. Additionally, 1.1% of subjects had epistaxis, and 4.3% had ethanolic intoxication (Table 3).

Out of the 352 subjects, 11.4% experienced coma, while 7.4% underwent orotracheal intubation and mechanical ventilation. Furthermore, 17 patients had negative outcomes related to cardiorespiratory arrest, with 3.1% being unresponsive to resuscitation maneuvers and 7.7% being responsive (Table 3).

Chest trauma is often associated with rib fractures, both with and without displacement. According to the literature, common complications include pneumothorax and hemopneumothorax. In our group, the prevalence of these complications was 8.5% of patients with pneumothorax and 2.6% of patients with hemopneumothorax (Table 3).

Abdominal trauma can also be complicated by pneumomediastinum (the presence of air in the mediastinum—the space located between the two lungs) or hemoperitoneum (free blood accumulated in the peritoneal cavity). In this study, we identified a low prevalence of these complications: 0.6% of patients had pneumomediastinum, and 3.4% had hemoperitoneum. Additionally, these injuries can be associated with visceral, often life-threatening, injuries such as ruptured spleen. The visceral injuries associated with organ ruptures identified in this group were spleen rupture in 3.44% of patients, liver rupture in 0.6% of patients, and liver and splenic rupture in 0.3% of patients (Table 3).

Furthermore, within this group, a significant number of subjects experienced traumatic shock (3.4% of patients), while 0.3% of patients experienced cardiogenic shock, and 0.6% of patients experienced hypovolemic shock. Additionally, 2.6% of patients experienced hemorrhagic shock, and 0.9% of patients experienced both traumatic shock and hemorrhagic shock (Table 3).

The odds ratio (OR) measures how strongly an event is associated with exposure. The odds ratio is a ratio of two sets of odds: events occurring in an exposed group versus those occurring in an unexposed group. In this study, to assess factors with a negative impact on multiple-trauma patients, we evaluated the probability of coma induction based on the presence of subarachnoid hemorrhage (SAH), pneumothorax, and hemopneumothorax.

The probability of presenting with subarachnoid hemorrhage (SAH) was 2.203 times higher in comatose patients than in non-comatose patients. Additionally, in patients who experienced SAH, the relative risk of coma induction was 2.053 compared to patients who did not experience SAH (0.932).

The probability of presenting with pneumothorax in comatose patients was 0.856 higher than in non-comatose patients. The relative risk of coma induction was 0.867 in patients who presented with pneumothorax compared to 1.013 in patients who did not (Table 4).

Hemopneumothorax was 2.293 times more likely to develop in comatose patients than in non-comatose patients. In addition, the relative risk supported these results. Specifically, the relative risk of coma induction was 2.229 times higher in patients with hemopneumothorax than in those without this complication (0.972) (Table 4) [17].

## 4. Discussion

There are no standard threshold values for multiple trauma, but mortality ranges from 10% in patients with an Injury Severity Score (ISS) of 15 to 20% in those with an ISS of >25. This results in 1 in 3 cases of severe multiple trauma resulting in severe disability, and the morbidity arising from such injuries is considerable [17,18].

There is a high incidence of multiple trauma in developing countries, and it continues to be one of the main causes of death among young people aged 10–40 years. In this study, the mean age of the subjects was 29.94 years, similar to other values found in the literature [19,20].

Trauma patients in rural areas are usually older, less severely injured, and more likely to die at the scene than urban patients. The rate of fatal accidents is more than twice as high in rural areas than in urban areas [21,22]. The most common mode of multiple trauma identified in this group was road traffic accidents, with a prevalence of 68.09%.

The outcome of patients with multiple trauma often depends on the severity of head injuries, affecting both short-term survival and long-term outcomes. Gennarelli demonstrated a continuous, progressive, inverse-proportional relationship between mortality following traumatic brain injury and GCS score. Specifically, they observed that the mortality rate increases as the GCS score decreases [23]. These results are supported by other studies in the literature [24]. The mean GCS score recorded in this group was 11.2, with most subjects presenting a GCS higher than 8 points.

Massive hemorrhages are the main cause of death in multiple-trauma patients. According to studies in the literature, one-third of trauma patients with major bleeding and almost half of all patients with massive bleeding will die [25]. Hemostasis requires a balance between coagulation and fibrinolysis, which allows the control of bleeding and the prevention of intravascular thrombosis. Major hemorrhage disrupts coagulum fibrinolysis, leading to altered hemostatic response and worsening blood loss [26]. In this study, we identified 2.6% of patients with hemorrhagic shock.

Normally, cellular and molecular interactions contribute to restoring tissue homeostasis and reducing acute inflammation [27]. Severe trauma is associated with systemic inflammatory syndrome. The endothelium activated by exposure to inflammatory cytokines becomes more porous, allowing mediators of tissue injury to access the intercellular space. This leads to a vicious circle of inflammation and immune pheresis, resulting in inflammation-associated sepsis and an increased risk of developing multi-organ dysfunction syndrome [23,28,29,30]. The first inflammatory response occurs immediately after injury, precipitating organ dysfunction in the days and weeks that follow [31,32]. The patient becomes vulnerable, and tissue hypoxia and hypovolemia set in, facilitating the onset of infection. The nature of the medical and surgical interventions required defines the second inflammatory moment. In this study, we identified a varied prevalence of different types of shock. Traumatic shock was found in 3.4% of subjects, whereas 0.3% of patients experienced cardiogenic shock, and 0.6% experienced hypovolemic shock. In addition, 0.9% of patients experienced both traumatic and hemorrhagic shock.

If early and adequate resuscitation following a major trauma fails, three key physiological disorders are found: hypothermia, coagulopathy, and acidosis. These are recognized in the literature as the “lethal triad” [1,31,33,34]. They exhibit a continuous effect on each other, ultimately resulting in patient death if not individually treated.

Multiple trauma is generally used to describe trauma patients whose injuries involve more than one region of the body, compromise the patient’s physiology, and may cause the dysfunction of uninjured organs [35]. Patients with multiple trauma are at increased risk of mortality due to the underlying pathophysiological response. According to the literature, head and brain injury and chest trauma are major risk factors in trauma patients, and the concomitant occurrence of these factors leads to an exponential increase in mortality [17,36,37]. Additionally, pulmonary contusions can cause decreased pulmonary reserve, leading to hypoventilation and hypoxia, subsequently causing secondary brain injury. Studies have also identified a significant correlation between early intubation and brain damage in patients with multiple trauma [38]. In our study, we identified that 11.4% of patients were in a coma, 7.4% of patients required OTI + MV, 3.1% of patients had an unresponsive CRA to resuscitation maneuvers, and 7.7% of patients were responsive to resuscitation maneuvers.

Injury to the abdomen can change nutritional balance and increase bacterial translocation from the gastrointestinal tract [39,40]. Increased levels of post-traumatic endotoxemia have also been reported in the literature [41].

The severity of injury, relevant pathophysiological changes, and physiological changes can be used for mortality prediction [35]. In addition, disorders produced in the post-traumatic immune system pose one of the greatest threats to life [42,43].

Severe multiple trauma often comes with traumatic intracranial hemorrhagic lesions. According to the literature, mortality rates increase about eightfold in cases involving both intracranial and extracranial hemorrhagic trauma (such as massive hemothorax, intra-abdominal organ injury, and pelvic fracture) compared to situations with just head trauma. In our study, we found a prevalence of 6.8% for SAH, 8.5% for pneumothorax, and 2.6% for hemopneumothorax. The severity of abdominal trauma was also evaluated based on the types of visceral injuries found in the group. We observed a prevalence of 3.44% for spleen rupture and 0.6% for liver rupture, with 0.3% of patients having both liver and spleen rupture [36]. In addition, the relative risk of inducting a coma was 2.053 times higher in patients with SAH, 0.867 times higher in patients with pneumothorax, and 2.229 times higher in patients with hemopneumothorax.

Head trauma is still a big problem worldwide. The rapid and comprehensive assessment of head injuries is crucial in managing each case because primary and secondary lesions can be threatening [44].

The prognosis largely depends on the time of presentation to the doctor but also on the patient’s comorbidities [45].

The goals of pharmacotherapy are to reduce morbidity, prevent complications, improve symptoms and quality of life, decrease hospitalizations, and improve mortality. The goal of pharmacologic therapy is to control symptoms and initiate and escalate drugs that reduce mortality and morbidity in multiple-trauma patients [46].

## 5. Conclusions

Factors that may influence the outcome of multiple-trauma patients include the severity of the initial injury, the number of injuries sustained, and the location of the injuries.

Patients with more severe injuries are at a higher risk of experiencing worse outcomes.

Additionally, patients with multiple injuries are also more likely to have worse outcomes.

Injuries to the head, neck, and spine are particularly serious and can result in life-threatening complications.

## Figures and Tables

**Table 1:** Prevalence of multiple trauma in the group by gender and year of case registration.

	Number of Cases	Percent
Gender		
Male	256	72.7%
Female	93	27.3%
Year		
2015	60	17.0%
2016	48	13.6%
2017	50	14.2%
2018	44	12.5%
2019	93	26.4%
2020	29	8.2%
2021	28	8.0%

**Table 2:** Type of identified multiple trauma mechanisms in the group.

Type of Multiple Trauma Mechanisms	Number of Patients	Percentage
Multiple trauma by aggression	24	7.29%
Multiple trauma by falling from height	51	15.50%
Multiple trauma by falling from the cart	6	1.82%
Multiple trauma by explosion/projection	6	1.82%
Multiple trauma incarcerated		0.61%
Multiple trauma by railway accident		0.61%
Multiple trauma by crushing	11	3.34%,
Multiple trauma by road traffic accident	224	64.09%
Multiple trauma by road traffic accident as pedestrian	3	0.91%

**Table 3:** Diagnoses and complications associated with multiple trauma in the studied group.

Diagnose	Number of Patients	Percentage	Complications	Number of Patients	Percentage
TBI/CFT			Hematoma		
Minor acute TBI, grade 0	47	13.4%	Epicranial	5	1.4%
Minor acute TBI, grade 1	25	7.1%	Subdural	17	4.8%
Minor acute TBI, grade 2	5	1.4%	Extradural	8	2.3%
Medium acute TBI	7	2.0%	Paraspinal	1	0.3%
Severe acute TBI	71	20.2%	Intraparenchymal	1	0.3%
TBI	35	9.9%	No	320	90.9%
CFT	18	5.1%	Coma		
TBI and CFT	4	1.1%	Yes	40	11.4%
No	140	39.8%	No	312	88.6%
OTI/MV			Epistaxis		
Yes	26	7.4%	Yes	4	1.1%
No	326	92.6%	No	348	98.9%
SAH			Ethanol intoxication		
Yes	24	6.8%	Yes	15	4.3%
No	328	93.2%	No	337	95.7%
SCR			Pneumothorax		
Nonresponsive to CPA	11	3.1%	Yes	30	8.5%
Resuscitated	6	1.7%	No	322	91.5%
Absent	335	95.2%	Pneumomediastinum		
Shock type			Yes	2	0.6%
Traumatic	12	3.4%	No	350	99.4%
Hemorrhagic	9	2.6%	Hemopneumothorax		
Traumatic and hemorrhagic	3	0.9%	Yes	9	2.6%
Cardiogenic	1	0.3%	No	343	97.4%
Hypovolemic	2	0.6%	Hemoperitoneum		
No	325	92.3%	Yes	12	3.4%
Organ rupture			No	340	96.6%
Spleen	12	3.4%			
Liver	2	0.6%			
Liver and spleen	1	0.3%			
No	337	95.7%			

**Table 4:** Estimated risk by associated pathologies of the group.

Coma/SAH *	***p* Value**	**95% Confidence Interval**	
		**Lower**	**Upper**
Odds Ratio for Coma (Yes/No)	2.203	0.774%	6.268%
For Cohort SAH = Yes	2.053	0.811%	5.195%
For Cohort SAH = No	0.932	0.826%	1.051%
Number of Valid Cases	352		
**Coma/Pneumothorax**	***p* Value**	**95% Confidence Interval**	
		**Lower**	**Upper**
Odds Ratio for Coma (Yes/No)	0.856	0.247%	2.960%
For Cohort Pneumothorax = Yes	0.867	0.275%	2.727%
For Cohort Pneumothorax = No	1.013	0.921%	1.113%
Number of Valid Cases	352		
**Coma/Hemopneumothorax**	***p* Value**	**95% Confidence Interval**	
		**Lower**	**Upper**
Odds Ratio for Coma (Yes/No)	2.293	0.460%	11.441%
For Cohort Hemopneumothorax = Yes	2.229	0.479%	10.360%
For Cohort Hemopneumothorax = No	0.972	0.903%	1.045%
Number of Valid Cases	352		

SAH *—Subarachnoid hemorrhage.

## Data Availability

Data is unavailable due to privacy or ethical restrictions, a statement is still required.

## Abbreviations

AIS	Abbreviated Injury Scale
ATLS	advanced trauma life support
CPA	cardiopulmonary arrest
CFT	craniofacial trauma
IIP	increased intracranial pressure
ISS	Injury Severity Score
GCS	Glasgow Coma Scale
Max	maximum
MV	mechanical ventilation
OTI	orotracheal intubation
SAH	subarachnoid hemorrhage
SD	standard deviation
TBI	traumatic brain injury
